# Association of Kidney Biopsy Needle Gauge with Postprocedure Complications and Biopsy Adequacy

**DOI:** 10.34067/KID.0000000835

**Published:** 2025-05-22

**Authors:** MaryKate Staunton, Kanika Garg, Sagar Sadarangani, Amrita Makhijani, Kyra Shelton, Emma Koval, Cathleen Liang, Melissa Shaw, Candice Kent, F. Perry Wilson, Chirag R. Parikh, Mark A. Perazella, Jeffrey Turner, Randy Luciano, Dennis G. Moledina

**Affiliations:** 1 University of Connecticut School of Medicine, Farmington, Connecticut; 2 Clinical and Translational Research Accelerator, Department of Internal Medicine, Yale School of Medicine, New Haven, Connecticut; 3 Philadelphia College of Osteopathic Medicine, Philadelphia, Pennsylvania; 4 Section of Nephrology, Department of Internal Medicine, Yale School of Medicine, New Haven, Connecticut; 5 Division of Nephrology, Internal Medicine, Johns Hopkins School of Medicine, Baltimore, Maryland

**Keywords:** complications, glomerular disease, renal biopsy

## Abstract

**Key Points:**

- Needle selection in kidney biopsy requires balancing risk of complications with benefit of obtaining diagnostic tissue.
- Our data show that higher needle gauge provides more tissue for diagnosis but might be associated with greater risk of complications.

**Background:**

While a larger needle gauge can provide additional kidney tissue for diagnosis, it might also predispose to greater complication risk. Here, we evaluate the safety and diagnostic adequacy of kidney biopsies in patients who underwent a biopsy with various needle sizes.

**Methods:**

We evaluated rates of biopsy-related complications and adequacy between participants who underwent biopsy with 16 versus 18 G needles. We assessed safety using a composite outcome of blood transfusion, angiographic intervention, hemoglobin drop of at least 2 g/dl, or at least a medium sized hematoma after kidney biopsy. We defined adequacy as the number of glomeruli available for diagnosis.

**Results:**

Among 781 participants included in the analysis, 665 biopsies (85%) were performed using a 16 G needle and 116 (15%) were performed using an 18 G needle. We observed similar odds of complications in the 16 G group as compared with the 18 G group in univariable analysis (odds ratio, 1.56 [95% confidence interval, 0.81 to 3.01]); however, there were higher odds of complications in the 16 G group in both multivariable analysis adjusting for prebiopsy bleeding risk factors (2.40 [1.16 to 4.93]) and propensity score weighted analysis (2.92 [1.28 to 6.67]). As compared with 18 G needle, 16 G biopsy needle was associated with more glomeruli obtained (13 [9–19] versus 11 [6–15], *P* < 0.001). The higher number of glomeruli in 16 G group persisted after multivariable adjustment (5.06 [2.52 to 7.59]) and propensity score weighted (4.39 [0.37 to 8.42]) analyses.

**Conclusions:**

Participants with kidney biopsies performed with a 16 G needle tended to sample more glomeruli and had similar complications on univariable analysis but higher complications when adjusting for prebiopsy risk factors. This indicates that larger needle gauge provided more tissue for diagnosis, but clinicians appropriately avoided its use in those at higher risk of complications. Our findings suggest that the use of a 16 G needle improves diagnostic yield but carries a higher adjusted risk of complications, underscoring the importance of individualized needle gauge selection.

## Visual Abstract

**Figure:**
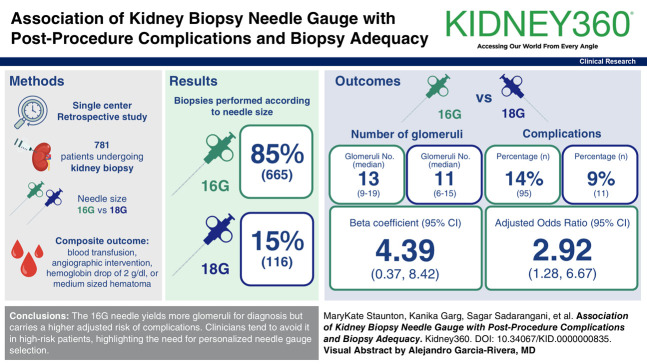

## Introduction

Percutaneous kidney biopsy is an important procedure for the diagnosis of various intrinsic kidney pathologies such as tubulointerstitial disease, vascular disease, or glomerular diseases.^1^ Many of the diagnoses made on a kidney biopsy are tied to specific treatment strategies and failure to diagnose these can lead to organ-threatening or life-threatening complications. While kidney biopsy is generally a safe procedure, a subset of patients experience bleeding complications that could be self-limiting such as drop in hemoglobin or perinephric hematoma or require interventions such as blood transfusion or surgical or radiologic interventions to stop bleeding, and, in rare cases, death.^1–7^

There is active ongoing research to identify various patient and procedure characteristics associated with postbiopsy complications in the hopes that careful selection of patients or mitigation of risk factors could prevent complications. Researchers, including our group, have found prebiopsy risk factors such as low platelets and low hemoglobin as tied to postbiopsy complications.^4,6^ Other known risk factors for postbiopsy complications include elevated international normalized ratio (INR) and use of antiplatelet and anticoagulation medications.^3,8^

Needle gauge selection poses a unique challenge in kidney biopsy procedure. While larger needle gauges sample more tissue for diagnosis, they simultaneously are also more likely to puncture blood vessels and therefore place patients at higher risk of complications. Some studies show that smaller needles could result in fewer postbiopsy complications, whereas others show that needles up to 16 G do not result in significantly more postbiopsy complications than smaller needles.^9–13^ Moreover, recent studies have found that choosing smaller gauge needles (18 G or smaller) could result in inadequate sampling for diagnosis.^9–12^ However, these observational studies did not control for prebiopsy likelihood of complications, which could have played a role in selection of needle gauge, and thus suffered from confounding by indication bias. To address this limitation, we tested the association of needle gauge with biopsy complications and diagnostic safety, adjusting for available prebiopsy risk factors using multivariable models and propensity-matched analysis.

## Methods

### Participants, Setting, and Study Design

In this study, we included participants enrolled in the Yale Kidney Biobank between January 2015 and April 2023. This cohort includes patients who underwent a percutaneous clinically indicated native kidney biopsy at either of the two Yale University-affiliated hospitals (York Street and St. Raphael's campuses of Yale New Haven Hospital). Patients who underwent a biopsy of a transplanted kidney or renal mass were excluded. We also excluded participants who underwent a kidney biopsy by interventional radiology due to differences in technique and population between radiology and nephrology. We excluded those in whom needle gauge was not known. All participants provided written informed consent for participation in the Kidney Biobank Cohort, and the activities were reviewed annually and approved by the Yale Human Investigation Committee. This study adhered to the Declaration of Helsinki.

### Outcomes and Predictors

Our primary complication outcome was a composite postbiopsy complication outcome that included need for blood transfusion related to biopsy complication, angiographic intervention to stop bleeding, at least a medium-sized hematoma after biopsy, and hemoglobin drop of ≥2 g/dl. We defined the presence of a hematoma as that which was at least 5 cm in any direction or described as moderate, medium, or large by the radiologist. Our primary adequacy outcome was the number of glomeruli available for analysis. Our primary exposure was the use of 16 G needle (versus 18 G). All data were obtained through review of electronic health records including biopsy reports and nephrology notes.

### Data Analysis

We present baseline characteristics as median (interquartile range) or count (percentage) compared between participants who underwent a kidney biopsy by 16 or 18 G needles. We used a chi-squared test for categorical variables and Wilcoxon rank-sum test for continuous variables. We tested the association of needle gauge with the composite complication outcome using a logistic regression model. We tested the association of number of glomeruli sampled versus needle gauge using linear regression. Model 1 was a univariable analysis; model 2 controlled for demographics (age, sex, and race) and laboratory features (hemoglobin, baseline eGFR, platelet count, INR, BUN, and creatinine at biopsy). Model 3 additionally controlled for procedural factors (inpatient, proceduralist training, desmopressin use, imaging modality, and number of passes) and prebiopsy and postbiopsy diagnosis. Model 4 used Inverse Probability of Treatment Weighting (IPTW) analysis, where we calculated propensity of being in each treatment group followed by weighting of observations as the inverse of the propensity score.^14^ This approach balanced key confounders between the two groups, as presented in Supplemental Table 1. Missingness in key variables is presented in Supplemental Table 2. For multivariable analysis, we replaced missing variables as either median for continuous or a separate category for categorical variables. For the propensity score analysis, missing covariate values were handled using the same approach to ensure that all key confounders were balanced between groups. All analysis were conducted in STATA version 18.0, and significance threshold was set at 0.05.

## Results

### Baseline Characteristics

**Figure 1:**
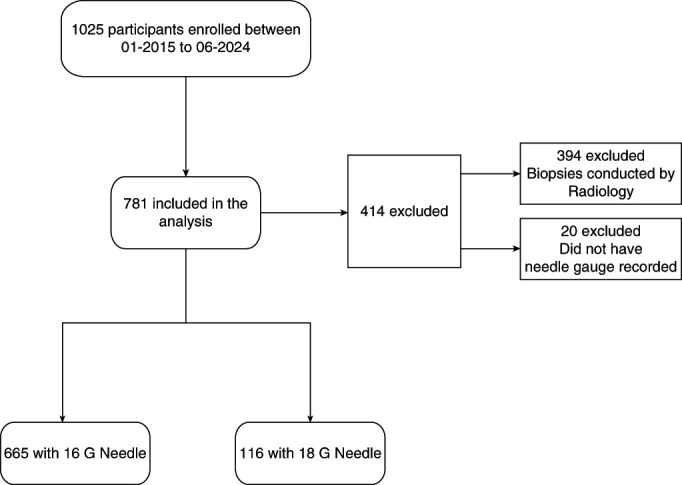
Standards for Reporting Diagnostic Accuracy Studies flow diagram of patients in the Yale biopsy cohort showing participants included in final analysis.

We included 781 participants who underwent kidney biopsies by at two Yale-affiliated hospitals (Figure 1). Of these, 665 (85%) were performed using a 16 G needle, and 116 (15%) were performed using an 18 G needle. Participants who underwent a biopsy using the 16 G needle tended to be younger (56 [42–68] versus 62 [57–74] years, *P* < 0.001), had a higher hemoglobin (11.2 [9.4–12.9] g/dl versus 10.2 [8.5–11.8], *P* < 0.001), and platelet count (240 [187–293] versus 199 [130–283] ×1000 per microliter, *P* < 0.001; Table 1). Furthermore, biopsies performed with the 16 G needle had lower occurrence of acute kidney disease, including AKI (29.0% versus 40.1%. *P* = 0.01) and lower serum creatinine (mg/dl; 2.00 [1.4–3.4] versus 2.24 [1.7–4.0], *P* < 0.001). Participants whose biopsies were performed with a 16 G needle also had higher number of passes (3 [2–3] versus 2 [2–3], *P* = 0.001).

**Table 1:** Baseline characteristics of study participants by needle gauge

Characteristic	16 G	18 G	*P* Value
Total	*N*=665	*N*=116	
Age (yr)	56 (42–67)	62 (51–72)	<0.001
Female, *n* (%)	321 (48)	42 (36)	0.02
Race/White, *n* (%)	385 (58)	77 (66)	0.09
BMI	28 (24–33)	28 (24–33)	0.93
Hypertension, *n* (%)	436 (66)	84 (72)	0.18
CKD, *n* (%)	181 (27)	29 (25)	0.43
Diabetes, *n* (%)	236 (36)	45 (39)	0.70
**Indications for biopsy, *n* (%)**			0.009
AKD or AKI	193 (31)	47 (44)	
Other	434 (69)	61 (57)	
**Features at biopsy**			
Baseline eGFR	35 (21–64)	30 (17–51)	0.04
Hemoglobin (g/dl)	11.20 (9.40–12.90)	10.15 (8.50–11.80)	<0.001
Hematocrit (%)	34.20 (29.00–39.10)	31.10 (26.00–36.60)	0.001
Platelet count (×1000 per microliter)	240 (187–293)	199 (130–283)	<0.001
INR	1.03 (0.97–1.14)	1.06 (0.97–1.30)	0.22
Serum creatinine (mg/dl)	2.00 (1.38–3.39)	2.24 (1.70–3.97)	0.01
BUN (mg/dl)	31 (21–53)	36 (26–53)	0.08
Inpatient, *n* (%)	216 (32)	47 (41)	0.09
**Procedure**			
No. of passes	3 (2–3)	2 (2–3)	0.001
Fellow, *n* (%)	143 (22)	36 (31)	0.03
Desmopressin use, *n* (%)	348 (52.5)	73 (62.9)	0.04
**Imaging type, *n* (%)**			0.003
CT-guided	163 (25)	26 (22)	
US-guided	502 (75)	90 (78)	

Data presented as *n* (%) for categorical variables or median (interquartile range) for continuous variables, the chi-squared test for categorical variables, and the Wilcoxon rank-sum test for continuous variable. AKD, acute kidney disease; BMI, body mass index; CT, computed tomography; INR, internalized normalized ratio; US, ultrasound.

### Comparison of Safety Outcomes between 16 and 18 G Needles

There were no significant differences in the composite safety outcomes or individual components of the composite outcome between biopsies done with 16 versus 18 G needles (Tables 2 and 3, model 1). However, multivariable analysis controlling for participant characteristics showed a significant association between 16 G needles with postbiopsy complications (2.19 [1.07 to 4.45], Table 3, model 2), which persisted (2.40 [1.16 to 4.93]; Table 3, model 3) after additionally controlling for procedural factors. Much of the increase in odds of complications in the 16 G needle group was due to higher odds of hematoma or hemoglobin drop (2.81 [1.21 to 6.54]) rather than need for blood transfusion or angiographic intervention (1.83 [0.65 to 5.14]). IPTW analysis showed similar results (Table 3, model 4).

**Table 2:** Comparison of kidney biopsy safety and adequacy outcomes between different needle gauges used for procedure

Characteristic	16 G Needle	18 G Needle	*P* Value
	*N*=665	*N*=116	
Composite safety outcome	95 (0.14)	11 (0.09)	0.16
Blood transfusion due to biopsy complication	22 (0.03)	5 (0.04)	0.61
Medium or large hematoma	34 (0.05)	4 (0.03)	0.42
Hemoglobin drop ≥2 g/dl	58 (0.10)	3 (0.03)	0.02
Angiographic intervention to stop bleeding	15 (0.02)	2 (0.02)	0.71
Transfusion or angiographic intervention	30 (0.05)	7 (0.06)	0.50
Hematoma or hemoglobin drop	82 (0.12)	7 (0.06)	0.05
Glomeruli obtained, *n*	13 (9–19)	11 (6–15)	<0.001

Data presented as *n* (%) or median (interquartile range), the chi-squared test for categorical variables, and the Wilcoxon rank-sum test for continuous variable.

**Table 3:** Independent association of needle gauge 16 with postbiopsy complications

Outcome	Model 1	Model 2	Model 3	Model 4
	OR (95% CI)^a^			
Composite safety outcome^a^	1.56 (0.81 to 3.01)	2.19 (1.07 to 4.45)^b^	2.40 (1.16 to 4.93)^b^	2.92 (1.28 to 6.67)^b^
Transfusion or IR intervention^a^	0.72 (0.31 to 1.69)	1.78 (0.65 to 4.86)	1.95 (0.70 to 5.44)	2.12 (0.74 to 6.03)
Hematoma or hemoglobin drop >2^a^	2.14 (0.96 to 4.77)	2.67 (1.14 to 6.22)^b^	2.88 (1.22 to 6.76)^b^	3.41 (1.23 to 9.44)^b^
	*β* coefficient (95% CI)^c^			
No. of glomeruli^c^	5.65 (3.22 to 8.08)^b^	5.26 (2.74 to 7.79)^b^	5.06 (2.52 to 7.59)^b^	4.39 (0.37 to 8.42)^b^

Hemoglobin change represents postbiopsy hemoglobin (lowest in 2 days after biopsy) prebiopsy hemoglobin (immediately before biopsy). Model 1, univariable. Model 2, controlling for demographics (age, sex, and race), laboratory features (hemoglobin, baseline eGFR, platelet count, international normalized ratio, BUN, and creatinine), and presence of cirrhosis. Model 3 additionally controls for procedural factors (imaging modality, desmopressin use, and location) and prebiopsy diagnosis. Missing covariate values replaced by median for continuous and with separate term for categorical. Model 4 is Inverse Probability of Treatment Weighting analysis. CI, confidence interval; IR, interventional radiology; OR, odds ratio.

a Indicates binary outcomes presented as odds ratios with 95% confidence intervals.

b *P* < 0.05.

c Indicates continuous outcome (number of glomeruli), presented as *β* coefficients with 95% confidence interval.

### Comparison of Adequacy Outcomes between 16 and 18 G Needles

**Figure 2:**
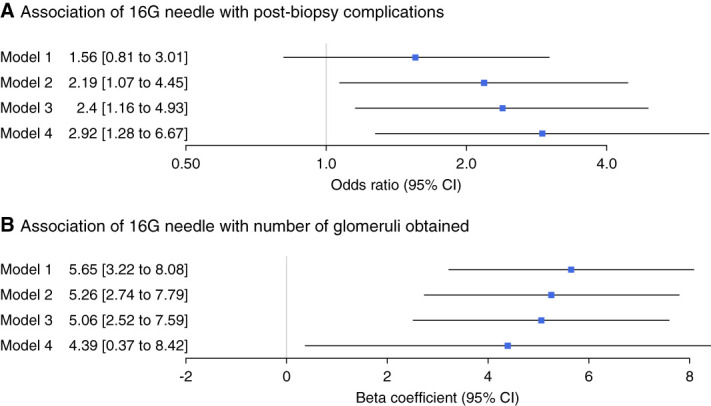
**Association of biopsy needle gauge with postbiopsy complications and number of glomeruli obtained.** Postbiopsy complication includes presence of hematoma, hemoglobin drop, need for blood transfusion, or angiographic intervention. Model 1, univariable. Model 2, controlling for demographics (age, sex, and race), laboratory features (hemoglobin, baseline eGFR, platelet count, INR, BUN, and creatinine), and presence of liver disease. Model 3 additionally controls for procedural factors (imaging modality, desmopressin use, and location) and prebiopsy diagnosis. Missing covariate values replaced by median for continuous and with separate term for categorical. Model 4 is Inverse Probability of Treatment Weighting analysis. CI, confidence interval; INR, internalized normalized ratio.

For the cohort, the 16 G biopsy needle was associated with more glomeruli obtained (13 [9–19] versus 11 [6–15]. *P* < 0.001; Table 2). A larger (16 G) needle was associated with more glomeruli obtained both in univariable analysis (*β* coefficient, 5.65; 95% confidence interval, 3.22 to 8.08) and on multivariable analysis controlling for demographics, laboratory features, and procedural factors (5.06 [2.52 to 7.59], *P* < 0.001). A trend toward higher glomeruli number in 16 G group was observed on IPTW analysis (4.39 [0.37 to 8.42], Figure 2).

## Discussion

Our data show that larger needle size was used in those at lower risk of bleeding after kidney biopsy, and although there were no differences in complications between various needle sizes on univariable analysis, larger needle size was associated with higher odds of complication in multivariable analysis controlling for these bleeding risk factors. These data indicate that clinicians appropriately avoided using larger needle size in those at higher risk of bleeding reducing the complication rate in these individuals to baseline. On the other hand, the use of larger needle size resulted in obtaining more glomeruli for diagnosis.

Selection of needle gauge for biopsy requires careful balancing of risk of bleeding outcomes versus obtaining more tissue for adequate sampling and diagnosis. Although percutaneous kidney biopsies are a relatively safe procedure,^3^ major and minor complications can occur in a subset of patients. However, there is no consensus on optimal balance of risk and benefit from the two commonly used needle sizes: 16 and 18 G. Studies have shown a higher risk of hematoma in patients whose biopsies were performed with a 16 G needle.^15,16^ On the other hand, 18 G needles result in inadequate samples, fragmented glomeruli, and fewer glomeruli.^9–11^ However, given the perception that smaller needle size is associated with fewer complications, clinicians might reserve these for those at higher risk of complications. Indeed, in our study, patients who had a biopsy performed using a 18 G needle had lower hemoglobin, hematocrit, and platelet counts while having higher serum creatinine levels. However, none of the above studies controlled for these patient-level risk factors for bleeding that could influence selection of needle gauge for biopsy.

In our analysis, we used multivariable models to control for prebiopsy risk factors that may influence both the selection of needle size as well as the outcome of biopsy complications. These included demographics (age, sex, and race), laboratory features (hemoglobin, baseline eGFR, platelet count, INR, BUN, and creatinine), prebiopsy and postbiopsy diagnosis, and procedural factors (proceduralist training, imaging modality, desmopressin use, and location). On univariable analysis, we noted no association of needle gauge with complications. However, in multivariable analysis, 16 G needle was associated with greater risk of biopsy complications. On the other hand, higher number of glomeruli obtained with a 16 G needle similar to other studies.^9,10^ Our cohort's results align with previous studies emphasizing the generally low—but non-negligible—risk of kidney biopsy complications and the need for individualized risk assessment, particularly in patients with comorbidities or altered hemostasis.^17,18^

Our findings provide interesting insights into current practice patterns regarding needle size choice and could influence choice of needle size for kidney biopsies to minimize the risk of repeat biopsies and improve safety outcomes. First, we noted that clinicians reserved smaller needle gauges that provided less tissue for diagnosis for those at higher risk of complication such that on unadjusted analysis, the risk of complication was similar between both needle sizes. This indicates that clinicians are aware of the risks and benefits of the two needle sizes and are appropriately balancing these for each patient. However, practice patterns differ across institutions, departments, and practitioners. Clinicians might use our data to avoid using larger needle size in those at higher risk of complications, while continuing to them in those with lower risk, particularly since the use of larger needle size yielded significantly more glomeruli for diagnosis. Our findings may also help inform researchers and potential participants of studies involved with research kidney biopsies, such as Kidney Precision Medicine Project, by providing risk estimates for protocol development and informed consent. Proper selection of needle gauge size for patients may improve quality of care while reducing risk for complications.

Our study has several strengths. We performed a chart review of all participants to establish their association with the biopsy. Furthermore, we systematically evaluated several risk factors to design a multivariable model. However, our study also has some limitations. First, this is a study spans two Yale-affiliated hospitals; the generalizability of our findings will need to be tested in a larger, multicenter study. There are also factors that can influence bleeding risk that were not included in analysis, such as resumption of anticoagulation and antiplatelet therapy after biopsy. In addition, as this is an observational study, we cannot say with certainty how needle gauge affect complication outcomes, and there are other complications such as gross hematuria which were not captured in this study. A key limitation is the lack of standardized prebiopsy coagulation testing. Coagulation laboratory results such as INR, activated partial thromboplastin time, and fibrinogen were not routinely obtained, and INR was missing in a substantial proportion of patients, limiting our ability to fully assess bleeding risk. Future studies can include more comprehensive patient factors and outcomes.

In conclusion, we found that biopsies were performed with a 16 G needle versus an 18 G needle had a higher number of glomeruli obtained but no significant difference in postbiopsy complications for patients in unadjusted analysis but greater odds of complications when accounting for preprocedural bleeding risk factors. This indicates that clinicians appropriately selected narrower needle gauge in patients at higher risk of complications, but this came at the cost of lower diagnostic yield. Ultimately, our findings indicate that the use of a 16 G needle improves diagnostic yield but carries a higher risk of complications, underscoring the importance of individualized needle gauge selection.

## Supplementary Material

## Data Availability

Partial restrictions to the data and/or mials apply. Data available upon reasonable request to the corresponding author.

## Supplemental Material

Supplemental Table 1. Standardized mean difference in key confounders before and after IPTW analysis.

Supplemental Table 2. Missingness of key covariates.

## References

[1] Luciano RL Moeckel GW. Update on the native kidney biopsy: core curriculum 2019. Am J Kidney Dis. 2019;73(3):404–415. doi:10.1053/j.ajkd.2018.10.011 30661724

[2] Poggio ED McClelland RL Blank KN, . Systematic review and meta-analysis of native kidney biopsy complications. Clin J Am Soc Nephrol. 2020;15(11):1595–1602. doi:10.2215/CJN.04710420 33060160 PMC7646247

[3] Lees JS Welsh CE Celis-Morales CA, . Glomerular filtration rate by differing measures, albuminuria and prediction of cardiovascular disease, mortality and end-stage kidney disease. Nat Med. 2019;25(11):1753–1760. doi:10.1038/s41591-019-0627-8 31700174 PMC6858876

[4] Aaltonen S Finne P Honkanen E. Outpatient kidney biopsy: a single center experience and review of literature. Nephron. 2020;144(1):14–20. doi:10.1159/000503255 31578024

[5] Korbet SM Gashti CN Evans JK Whittier WL. Risk of percutaneous renal biopsy of native kidneys in the evaluation of acute kidney injury. Clin Kidney J. 2018;11(5):610–615. doi:10.1093/ckj/sfy048 30289129 PMC6165762

[6] Moledina DG Luciano RL Kukova L, . Kidney biopsy-related complications in hospitalized patients with acute kidney disease. Clin J Am Soc Nephrol. 2018;13(11):1633–1640. doi:10.2215/CJN.04910418 30348813 PMC6237071

[7] Whittier WL Sayeed K Korbet SM. Clinical factors influencing the decision to transfuse after percutaneous native kidney biopsy. Clin Kidney J. 2016;9(1):102–107. doi:10.1093/ckj/sfv128 26798469 PMC4720206

[8] Corapi KM Chen JL Balk EM Gordon CE. Bleeding complications of native kidney biopsy: a systematic review and meta-analysis. Am J Kidney Dis. 2012;60(1):62–73. doi:10.1053/j.ajkd.2012.02.330 22537423

[9] Sousanieh G Whittier WL Rodby RA Peev V Korbet SM. Percutaneous renal biopsy using an 18-gauge automated needle is not optimal. Am J Nephrol. 2020;51(12):982–987. doi:10.1159/000512902 33454708

[10] Mai J Yong J Dixson H, . Is bigger better? A retrospective analysis of native renal biopsies with 16 Gauge versus 18 Gauge automatic needles. Nephrology (Carlton). 2013;18(7):525–530. doi:10.1111/nep.12093 23639213

[11] Roth R Parikh S Makey D, . When size matters: diagnostic value of kidney biopsy according to the gauge of the biopsy needle. Am J Nephrol. 2013;37(3):249–254. doi:10.1159/000347219 23485619

[12] Peters B Mölne J Hadimeri H Hadimeri U Stegmayr B. Sixteen Gauge biopsy needles are better and safer than 18 Gauge in native and transplant kidney biopsies. Acta Radiol. 2017;58(2):240–248. doi:10.1177/0284185116641349 27055922

[13] Chunduri S Whittier WL Korbet SM. Adequacy and complication rates with 14- vs. 16-gauge automated needles in percutaneous renal biopsy of native kidneys. Semin Dial. 2015;28(2):E11–E14. doi:10.1111/sdi.12332 25441680

[14] Chesnaye NC Stel VS Tripepi G, . An introduction to inverse probability of treatment weighting in observational research. Clin Kidney J. 2022;15(1):14–20. doi:10.1093/ckj/sfab158 35035932 PMC8757413

[15] Cui S Heller HT Waikar SS McMahon GM. Needle size and the risk of kidney biopsy bleeding complications. Kidney Int Rep. 2016;1(4):324–326. doi:10.1016/j.ekir.2016.08.017 29142935 PMC5678857

[16] Simard-Meilleur MC Troyanov S Roy L Dalaire E Brachemi S. Risk factors and timing of native kidney biopsy complications. Nephron Extra. 2014;4(1):42–49. doi:10.1159/000360087 24803920 PMC4000304

[17] Koirala A Jefferson JA. How safe is a native kidney biopsy? Clin J Am Soc Nephrol. 2020;15(11):1541–1542. doi:10.2215/CJN.14890920 33060161 PMC7646248

[18] Halimi JM Gatault P Longuet H, . Major bleeding and risk of death after percutaneous native kidney biopsies: a French nationwide cohort study. Clin J Am Soc Nephrol. 2020;15(11):1587–1594. doi:10.2215/CJN.14721219 33060158 PMC7646233

