# Supplementary material for: Association of Kidney Biopsy Needle Gauge with Postprocedure Complications and Biopsy Adequacy
Source: Kidney360. 2025 May 22;6(10):1682–8. doi: 10.34067/KID.0000000835 (PMC12778012; doi:10.34067/KID.0000000835)
Supplement: Supplementary file 1 [file kidney360-6-1682-s001.pdf]

ASN American Society of Nephrology | JASN Journal of the American Society of Nephrology | KIDNEY360 Accessing Our World From Every Angle | CJASN Clinical Journal of the American Society of Nephrology

ASN Journal Disclosure Form

As per ASN journal policy, I have disclosed any financial relationships or commitments I have held in the past 36 months as included below. I have listed my Current Employer below to indicate there is a relationship requiring disclosure. If no relationship exists, my Current Employer is not listed.

K. Garg has nothing to disclose.

I understand that the information above will be published within the journal article, if accepted, and that failure to comply and/or to accurately and completely report the potential financial conflicts of interest could lead to the following: 1) Prior to publication, article rejection, or 2) Post-publication, sanctions ranging from, but not limited to, issuing a correction, reporting the inaccurate information to the authors' institution, banning authors from submitting work to ASN journals for varying lengths of time, and/or retraction of the published work.

Name: Kanika K. Garg
Manuscript ID: 9325802b8c91c867
Manuscript Title: Association of Kidney Biopsy Needle Gauge with Post-Procedure Complications and Biopsy Adequacy
Date of Completion: May 1, 2025
Disclosure Updated Date: May 1, 2025

ASN American Society of Nephrology | JASN Journal of the American Society of Nephrology | KIDNEY360 Accessing Our World From Every Angle | CJASN Clinical Journal of the American Society of Nephrology

ASN Journal Disclosure Form

As per ASN journal policy, I have disclosed any financial relationships or commitments I have held in the past 36 months as included below. I have listed my Current Employer below to indicate there is a relationship requiring disclosure. If no relationship exists, my Current Employer is not listed.

C. Kent reports the following:
Employer: Yale University

I understand that the information above will be published within the journal article, if accepted, and that failure to comply and/or to accurately and completely report the potential financial conflicts of interest could lead to the following: 1) Prior to publication, article rejection, or 2) Post-publication, sanctions ranging from, but not limited to, issuing a correction, reporting the inaccurate information to the authors' institution, banning authors from submitting work to ASN journals for varying lengths of time, and/or retraction of the published work.

Name: Candice Kent
Manuscript ID: K360-2025-000161R1
Manuscript Title: Association of Kidney Biopsy Needle Gauge with Post-Procedure Complications and Biopsy Adequacy
Date of Completion: April 21, 2025
Disclosure Updated Date: February 3, 2025

ASN American Society of Nephrology | JASN Journal of the American Society of Nephrology | KIDNEY360 Accessing Our World From Every Angle | CJASN Clinical Journal of the American Society of Nephrology

ASN Journal Disclosure Form

As per ASN journal policy, I have disclosed any financial relationships or commitments I have held in the past 36 months as included below. I have listed my Current Employer below to indicate there is a relationship requiring disclosure. If no relationship exists, my Current Employer is not listed.

E. Koval has nothing to disclose.

I understand that the information above will be published within the journal article, if accepted, and that failure to comply and/or to accurately and completely report the potential financial conflicts of interest could lead to the following: 1) Prior to publication, article rejection, or 2) Post-publication, sanctions ranging from, but not limited to, issuing a correction, reporting the inaccurate information to the authors' institution, banning authors from submitting work to ASN journals for varying lengths of time, and/or retraction of the published work.

Name: Emma L Koval
Manuscript ID: K360-2025-000161R1
Manuscript Title: Association of Kidney Biopsy Needle Gauge with Post-Procedure Complications and Biopsy Adequacy
Date of Completion: April 22, 2025
Disclosure Updated Date: April 22, 2025

ASN American Society of Nephrology
JASN Journal of the American Society of Nephrology
KIDNEY360 Accessing Our World From Every Angle
CJASN Clinical Journal of the American Society of Nephrology

ASN Journal Disclosure Form

As per ASN journal policy, I have disclosed any financial relationships or commitments I have held in the past 36 months as included below. I have listed my Current Employer below to indicate there is a relationship requiring disclosure. If no relationship exists, my Current Employer is not listed.

C. Liang has nothing to disclose.

I understand that the information above will be published within the journal article, if accepted, and that failure to comply and/or to accurately and completely report the potential financial conflicts of interest could lead to the following: 1) Prior to publication, article rejection, or 2) Post-publication, sanctions ranging from, but not limited to, issuing a correction, reporting the inaccurate information to the authors' institution, banning authors from submitting work to ASN journals for varying lengths of time, and/or retraction of the published work.

Name: Cathleen G Liang
Manuscript ID: K360-2025-000161R1
Manuscript Title: Association of Kidney Biopsy Needle Gauge with Post-Procedure Complications and Biopsy Adequacy
Date of Completion: April 22, 2025
Disclosure Updated Date: April 22, 2025

ASN
American Society of Nephrology
JASN
Journal of the American Society of Nephrology
KIDNEY360
Accessing Our World From Every Angle
CJASN
Clinical Journal of the American Society of Nephrology

ASN Journal Disclosure Form

Date: 5/1/2025

Author: Randy Luciano

Manuscript ID: K360-2025-000161R1

Manuscript Title: Association of Kidney Biopsy Needle Gauge with Post-Procedure Complications and Biopsy Adequacy

Disclosure Statements

Nothing to disclose

ASN Journal Disclosure Form

As per ASN journal policy, I have disclosed any financial relationships or commitments I have held in the past 36 months as included below. I have listed my Current Employer below to indicate there is a relationship requiring disclosure. If no relationship exists, my Current Employer is not listed.

A. Makhijani reports the following:
Employer: Yale School of Medicine

I understand that the information above will be published within the journal article, if accepted, and that failure to comply and/or to accurately and completely report the potential financial conflicts of interest could lead to the following: 1) Prior to publication, article rejection, or 2) Post-publication, sanctions ranging from, but not limited to, issuing a correction, reporting the inaccurate information to the authors' institution, banning authors from submitting work to ASN journals for varying lengths of time, and/or retraction of the published work.

Name: Amrita Makhijani
Manuscript ID: K360-2025-000161R1
Manuscript Title: Association of Kidney Biopsy Needle Gauge with Post-Procedure Complications and Biopsy Adequacy
Date of Completion: April 25, 2025
Disclosure Updated Date: April 25, 2025

ASN American Society of Nephrology JASN Journal of the American Society of Nephrology KIDNEY360 Accessing Our World From Every Angle CJASN Clinical Journal of the American Society of Nephrology

ASN Journal Disclosure Form

As per ASN journal policy, I have disclosed any financial relationships or commitments I have held in the past 36 months as included below. I have listed my Current Employer below to indicate there is a relationship requiring disclosure. If no relationship exists, my Current Employer is not listed.

D. Moledina reports the following:
Employer: Yale University School of Medicine; Consultancy: BioHaven, Inc.; Ownership Interest: Predict AIN, LLC; Research Funding: NIDDK; Honoraria: Healthcentral; Patents or Royalties: DGM is a coinventor of the pending patent application "Methods and Systems for Diagnosis of Acute Interstitial Nephritis"; and Advisory or Leadership Role: ASN journals, editorial board member.

I understand that the information above will be published within the journal article, if accepted, and that failure to comply and/or to accurately and completely report the potential financial conflicts of interest could lead to the following: 1) Prior to publication, article rejection, or 2) Post-publication, sanctions ranging from, but not limited to, issuing a correction, reporting the inaccurate information to the authors' institution, banning authors from submitting work to ASN journals for varying lengths of time, and/or retraction of the published work.

Name: Dennis G. Moledina
Manuscript ID: K360-2025-000161R1
Manuscript Title: Association of Kidney Biopsy Needle Gauge with Post-Procedure Complications and Biopsy Adequacy
Date of Completion: April 21, 2025
Disclosure Updated Date: February 3, 2025

ASN American Society of Nephrology
JASN Journal of the American Society of Nephrology
KIDNEY360 Accessing Our World From Every Angle
CJASN Clinical Journal of the American Society of Nephrology

ASN Journal Disclosure Form

As per ASN journal policy, I have disclosed any financial relationships or commitments I have held in the past 36 months as included below. I have listed my Current Employer below to indicate there is a relationship requiring disclosure. If no relationship exists, my Current Employer is not listed.

C. Parikh reports the following:
Employer: Johns Hopkins University School of Medicine; Ownership Interest: Renalytix; Research Funding: National Institute of Diabetes and Digestive and Kidney Diseases (NIDDK); National Heart, Lung and Blood Institute (NHLBI); AstraZeneca; Alexion; and Advisory or Leadership Role: AstraZeneca; Alexion; Panoramic Science.

I understand that the information above will be published within the journal article, if accepted, and that failure to comply and/or to accurately and completely report the potential financial conflicts of interest could lead to the following: 1) Prior to publication, article rejection, or 2) Post-publication, sanctions ranging from, but not limited to, issuing a correction, reporting the inaccurate information to the authors' institution, banning authors from submitting work to ASN journals for varying lengths of time, and/or retraction of the published work.

Name: Chirag R. Parikh
Manuscript ID: K360-2025-000161R1
Manuscript Title: Association of Kidney Biopsy Needle Gauge with Post-Procedure Complications and Biopsy Adequacy
Date of Completion: April 23, 2025
Disclosure Updated Date: April 23, 2025

ASN American Society of Nephrology JASN Journal of the American Society of Nephrology KIDNEY360 Accessing Our World From Every Angle CJASN Clinical Journal of the American Society of Nephrology

ASN Journal Disclosure Form

As per ASN journal policy, I have disclosed any financial relationships or commitments I have held in the past 36 months as included below. I have listed my Current Employer below to indicate there is a relationship requiring disclosure. If no relationship exists, my Current Employer is not listed.

M. Perazella reports the following:
Employer: Yale School of Medicine; Honoraria: UpToDate; and Advisory or Leadership Role: Kidney360 (Deputy Editor), Journal of Ono-Nephrology (Co-Editor-in-Chief), Clinical Nephrology (AKI Series Editor), CJASN, KI, and KI Reports (Editorial Board).

I understand that the information above will be published within the journal article, if accepted, and that failure to comply and/or to accurately and completely report the potential financial conflicts of interest could lead to the following: 1) Prior to publication, article rejection, or 2) Post-publication, sanctions ranging from, but not limited to, issuing a correction, reporting the inaccurate information to the authors' institution, banning authors from submitting work to ASN journals for varying lengths of time, and/or retraction of the published work.

Name: Mark A. Perazella
Manuscript ID: K360-2025-000161R1
Manuscript Title: Association of Kidney Biopsy Needle Gauge with Post-Procedure Complications and Biopsy Adequacy
Date of Completion: April 21, 2025
Disclosure Updated Date: January 7, 2025

ASN American Society of Nephrology
JASN Journal of the American Society of Nephrology
KIDNEY360 Accessing Our World From Every Angle
CJASN Clinical Journal of the American Society of Nephrology

ASN Journal Disclosure Form

As per ASN journal policy, I have disclosed any financial relationships or commitments I have held in the past 36 months as included below. I have listed my Current Employer below to indicate there is a relationship requiring disclosure. If no relationship exists, my Current Employer is not listed.

S. Sadarangani has nothing to disclose.

I understand that the information above will be published within the journal article, if accepted, and that failure to comply and/or to accurately and completely report the potential financial conflicts of interest could lead to the following: 1) Prior to publication, article rejection, or 2) Post-publication, sanctions ranging from, but not limited to, issuing a correction, reporting the inaccurate information to the authors' institution, banning authors from submitting work to ASN journals for varying lengths of time, and/or retraction of the published work.

Name: Sagar S. Sadarangani
Manuscript ID: K360-2025-000161R1
Manuscript Title: Association of Kidney Biopsy Needle Gauge with Post-Procedure Complications and Biopsy Adequacy
Date of Completion: April 21, 2025
Disclosure Updated Date: October 16, 2024

ASN
American Society of Nephrology
JASN
Journal of the American Society of Nephrology
KIDNEY360
Accessing Our World From Every Angle
CJASN
Clinical Journal of the American Society of Nephrology

ASN Journal Disclosure Form

As per ASN journal policy, I have disclosed any financial relationships or commitments I have held in the past 36 months as included below. I have listed my Current Employer below to indicate there is a relationship requiring disclosure. If no relationship exists, my Current Employer is not listed.

M. Shaw reports the following:
Employer: Yale

I understand that the information above will be published within the journal article, if accepted, and that failure to comply and/or to accurately and completely report the potential financial conflicts of interest could lead to the following: 1) Prior to publication, article rejection, or 2) Post-publication, sanctions ranging from, but not limited to, issuing a correction, reporting the inaccurate information to the authors' institution, banning authors from submitting work to ASN journals for varying lengths of time, and/or retraction of the published work.

Name: Melissa M. Shaw
Manuscript ID: K360-2025-000161R1
Manuscript Title: Association of Kidney Biopsy Needle Gauge with Post-Procedure Complications and Biopsy Adequacy
Date of Completion: April 21, 2025
Disclosure Updated Date: February 25, 2025

ASN American Society of Nephrology JASN Journal of the American Society of Nephrology KIDNEY360 Accessing Our World From Every Angle CJASN Clinical Journal of the American Society of Nephrology

ASN Journal Disclosure Form

As per ASN journal policy, I have disclosed any financial relationships or commitments I have held in the past 36 months as included below. I have listed my Current Employer below to indicate there is a relationship requiring disclosure. If no relationship exists, my Current Employer is not listed.

K. Shelton reports the following:
Employer: Clinical & Translational Research Accelerator

I understand that the information above will be published within the journal article, if accepted, and that failure to comply and/or to accurately and completely report the potential financial conflicts of interest could lead to the following: 1) Prior to publication, article rejection, or 2) Post-publication, sanctions ranging from, but not limited to, issuing a correction, reporting the inaccurate information to the authors' institution, banning authors from submitting work to ASN journals for varying lengths of time, and/or retraction of the published work.

Name: Kyra A. Shelton
Manuscript ID: K360-2025-000161R1
Manuscript Title: Association of Kidney Biopsy Needle Gauge with Post-Procedure Complications and Biopsy Adequacy
Date of Completion: April 21, 2025
Disclosure Updated Date: April 21, 2025

ASN American Society of Nephrology | JASN Journal of the American Society of Nephrology | KIDNEY360 Accessing Our World From Every Angle | CJASN Clinical Journal of the American Society of Nephrology

ASN Journal Disclosure Form

As per ASN journal policy, I have disclosed any financial relationships or commitments I have held in the past 36 months as included below. I have listed my Current Employer below to indicate there is a relationship requiring disclosure. If no relationship exists, my Current Employer is not listed.

M. Staunton reports the following:
Employer: University of Connecticut School of Medicine

I understand that the information above will be published within the journal article, if accepted, and that failure to comply and/or to accurately and completely report the potential financial conflicts of interest could lead to the following: 1) Prior to publication, article rejection, or 2) Post-publication, sanctions ranging from, but not limited to, issuing a correction, reporting the inaccurate information to the authors' institution, banning authors from submitting work to ASN journals for varying lengths of time, and/or retraction of the published work.

Name: Mary Kate Staunton
Manuscript ID: K360-2025-000161R1
Manuscript Title: "Association of Kidney Biopsy Needle Gauge with Post-Procedure Complications and Biopsy Adequacy."
Date of Completion: April 20, 2025
Disclosure Updated Date: April 20, 2025

ASN American Society of Nephrology | JASN Journal of the American Society of Nephrology | KIDNEY360 Accessing Our World From Every Angle | CJASN Clinical Journal of the American Society of Nephrology

ASN Journal Disclosure Form

As per ASN journal policy, I have disclosed any financial relationships or commitments I have held in the past 36 months as included below. I have listed my Current Employer below to indicate there is a relationship requiring disclosure. If no relationship exists, my Current Employer is not listed.

J. Turner reports the following:
Employer: Yale University; Consultancy: Sequana Medical; and Research Funding: Sequana Medical, Bayer, AstraZeneca.

I understand that the information above will be published within the journal article, if accepted, and that failure to comply and/or to accurately and completely report the potential financial conflicts of interest could lead to the following: 1) Prior to publication, article rejection, or 2) Post-publication, sanctions ranging from, but not limited to, issuing a correction, reporting the inaccurate information to the authors' institution, banning authors from submitting work to ASN journals for varying lengths of time, and/or retraction of the published work.

Name: Jeffrey M. Turner
Manuscript ID: K360-2025-000161R1
Manuscript Title: Association of Kidney Biopsy Needle Gauge with Post-Procedure Complications and Biopsy Adequacy
Date of Completion: April 21, 2025
Disclosure Updated Date: May 21, 2024

ASN American Society of Nephrology | JASN Journal of the American Society of Nephrology | KIDNEY360 Accessing Our World From Every Angle | CJASN Clinical Journal of the American Society of Nephrology

ASN Journal Disclosure Form

As per ASN journal policy, I have disclosed any financial relationships or commitments I have held in the past 36 months as included below. I have listed my Current Employer below to indicate there is a relationship requiring disclosure. If no relationship exists, my Current Employer is not listed.

F. Wilson reports the following:
Employer: Yale School of Medicine; Consultancy: Hekaheart; Aura.Care; WndrHLTH; Ownership Interest: Owner of Efference, LLC; Research Funding: Amgen; Vifor; Whoop; Advisory or Leadership Role: Editorial Board - American Journal of Kidney Disease; Editorial Board - Clinical Journal of the American Society of Nephrology; and Other Interests or Relationships: Medical columnist- Medscape.

I understand that the information above will be published within the journal article, if accepted, and that failure to comply and/or to accurately and completely report the potential financial conflicts of interest could lead to the following: 1) Prior to publication, article rejection, or 2) Post-publication, sanctions ranging from, but not limited to, issuing a correction, reporting the inaccurate information to the authors' institution, banning authors from submitting work to ASN journals for varying lengths of time, and/or retraction of the published work.

Name: Francis Perry Wilson
Manuscript ID: K360-2025-000161R1
Manuscript Title: "Association of Kidney Biopsy Needle Gauge with Post-Procedure Complications and Biopsy Adequacy
Date of Completion: April 21, 2025
Disclosure Updated Date: August 1, 2024
