# Supplementary material for: Association of Kidney Biopsy Needle Gauge with Postprocedure Complications and Biopsy Adequacy
Source: Kidney360. 2025 May 22;6(10):1682–8. doi: 10.34067/KID.0000000835 (PMC12778012; doi:10.34067/KID.0000000835)
Supplement: Supplementary file 2 [file kidney360-6-1682-s002.pdf]

Association of Kidney Biopsy Needle Gauge with Post-Procedure Complications and Biopsy Adequacy

Staunton and Garg et al

Contents

Supplemental Table 1. Standardized mean difference in key confounders before and after inverse probability of treatment weights (IPTW) analysis 2

Supplemental Table 2. Missingness of key covariates 3

Supplemental Table 1. Standardized mean difference in key confounders before and after inverse probability of treatment weights (IPTW) analysis

Characteristic	SMD before IPTW	SMD after IPTW
**Age**	-0.43	-0.16
**Male sex**	-0.25	0.03
**Race**	0.02	0.01
**eGFR**	0.21	0.06
**Hemoglobin**	0.36	0.08
**Platelet**	0.27	-0.05
**INR**	-0.34	0.02
**Serum creatinine**	-0.14	-0.09
**BUN**	-0.11	-0.02
**Cirrhosis**	-0.43	-0.02
**Inpatient**	-0.17	-0.07
**Trainee**	-0.22	-0.08
**CT guided**	0.05	-0.03
**Desmopressin**	-0.15	-0.08
**Passes**	0.34	0.01
**Pre-biopsy AIN or ATI**	-0.26	-0.07

SMD, standardized mean difference

Supplemental Table 2. Missingness of key covariates

Characteristic	Missing
Total	0
Age (years)	0
Female	2
Race/White	0
Body mass index (BMI)	12
Hypertension	5
Chronic Kidney Disease (CKD)	0
Diabetes	6
Acute Kidney Disease (AKD) or Acute Kidney Injury (AKI)	46
Baseline estimated glomerular filtration rate (eGFR)	13
Hemoglobin (g/dL)	6
Hematocrit (%)	14
Platelet count (*1000 per µL)	6
Internalized normalized ratio (INR)	525
Serum creatinine (mg/dL)	0
Blood urea nitrogen (BUN) (mg/dL)	10
Inpatient	0
# of passes	16
Fellow	5
Desmopressin use, n (%)	73
CT-guided	0
US-guided	0
